# Evaluating Propensity Score Methods in a Quasi-Experimental Study of the Impact of Menu-Labeling

**DOI:** 10.1371/journal.pone.0144962

**Published:** 2015-12-17

**Authors:** Stephanie L. Mayne, Brian K. Lee, Amy H. Auchincloss

**Affiliations:** Department of Epidemiology and Biostatistics, School of Public Health, Drexel University, Philadelphia, Pennsylvania, United States of America; Arizona State University, UNITED STATES

## Abstract

**Background:**

Quasi-experimental studies of menu labeling have found mixed results for improving diet. Differences between experimental groups can hinder interpretation. Propensity scores are an increasingly common method to improve covariate balance, but multiple methods exist and the improvements associated with each method have rarely been compared. In this re-analysis of the impact of menu labeling, we compare multiple propensity score methods to determine which methods optimize balance between experimental groups.

**Methods:**

Study participants included adult customers who visited full-service restaurants with menu labeling (treatment) and without (control). We compared the balance between treatment groups obtained by four propensity score methods: 1) 1:1 nearest neighbor matching (NN), 2) augmented 1:1 NN (using caliper of 0.2 and an exact match on an imbalanced covariate), 3) full matching, and 4) inverse probability weighting (IPW). We then evaluated the treatment effect on differences in nutrients purchased across the different methods.

**Results:**

1:1 NN resulted in worse balance than the original unmatched sample (average standardized absolute mean distance [ASAM]: 0.185 compared to 0.171). Augmented 1:1 NN improved balance (ASAM: 0.038) but resulted in a large reduction in sample size. Full matching and IPW improved balance over the unmatched sample without a reduction in sample size (ASAM: 0.049 and 0.031, respectively). Menu labeling was associated with decreased calories, fat, sodium and carbohydrates in the unmatched analysis. Results were qualitatively similar in the propensity score matched/weighted models.

**Conclusions:**

While propensity scores offer an increasingly popular tool to improve causal inference, choosing the correct method can be challenging. Our results emphasize the benefit of examining multiple methods to ensure results are consistent, and considering approaches beyond the most popular method of 1:1 NN matching.

## Introduction

Changes to diet, particularly an increase in energy intake, have been implicated as a major cause of the rising obesity epidemic.[1, 2] Food prepared away from home is typically higher in calories and lower in nutrients than food prepared at home, yet comprises approximately one-third of all calories purchased in the United States.[3] As of 2008, many municipal and state governments have mandated that chain restaurants display nutrition information at point of purchase. The regulations aim to promote nutrition awareness which may increase healthier offerings and choices among restaurants and customers, respectively. However, quasi-experimental studies evaluating the effectiveness of these policies have reported mixed results.[4–9] One of the few studies to find a labeling impact was Auchincloss et al (2013) which studied the impact of the city of Philadelphia’s menu-labeling ordinance.[10] The study found that customers at full-service restaurants with labeling purchased fewer calories, sodium, and saturated fat compared to customers at restaurants without labeling.[5] However, the cross-sectional study design was suboptimal and there was imperfect covariate balance between customers who dined at labeled versus unlabeled restaurants. Imbalance can lead to selection bias, in which those who have the opportunity to be exposed to the policy differ from those who do not on certain measured or unmeasured characteristics related to the outcome of interest.

In this study, we re-analyze data from Auchincloss et al (2013) via propensity score methods and compare the effect of menu labeling on nutritional outcomes, including purchased calories, saturated fat, sodium, and carbohydrates. The objectives of the study were to determine which method resulted in optimal balance between study arms and to determine if the association between menu labeling and nutritional outcomes was similar across methods.

Matching or weighting on propensity scores, defined as the odds or probability of receiving treatment based on a set of covariates,[11] are commonly used methods to reduce the imbalance of covariates between treatment groups. Propensity scores provide a balancing measure that can be used to match treated and untreated individuals based on their probability of being exposed to the intervention, and can also be used as weights or to stratify the study sample. Propensity score matching and weighting have been proposed as alternatives to multivariable regression models to address imbalance between groups in natural experiments,[12] and these approaches have been used in several previously published studies of responses to policy or built environment changes using quasi-experimental designs.[13–15] However, the relative performance of the different methods available has infrequently been compared, in settings that may not generalize to all observational study contexts.[16, 17]

## Methods

### Sampling

This study is a secondary analysis of a previously published study.[5] One large, mid-priced national chain restaurant was selected for inclusion in the study because it had a sufficient number of outlets in Philadelphia, where menu labeling was mandated, and outside Philadelphia, where no menu labeling was required. A convenience sample was included with two outlets in Philadelphia (case restaurants) and five outlets from within a 130-mile radius outside of the city (control restaurants). Menus were identical between treatment and comparison restaurants except that at case restaurants, nutrition information was clearly displayed in labels next to all food items, which included calories, grams of saturated fat, trans fat, carbohydrates, and milligrams of sodium.

### Data collection

Data sources included receipts and surveys collected from customers exiting the restaurants between 6-9pm on Sundays and Tuesdays-Thursdays over a one-month period in August, 2011. All data were anonymous with no identifiers. Surveys were used to obtain information on whether respondents saw nutrition information and whether this information affected what they ordered, demographic characteristics, frequency of dining at chain restaurants, and whether a health professional had recommended limiting calories, fat, carbohydrates and sodium. Study staff recorded participants’ body size (thin/average, overweight, and severely overweight) and whether the party included children.

### Outcome

The primary outcomes were number of calories purchased from food and beverages combined, number of food calories, grams of saturated fat, grams of carbohydrates, and milligrams of sodium in food purchases.

### Covariates

Covariates included age, gender, race, education, income, body size, whether a health professional cautioned the participant about his/her diet, frequency of dining at chain restaurants, day of the week, whether there were children in the party, and whether the customer made any substitutions or customizations to their order. Day of the week, the presence of children in the party, and whether substitutions were made were included as selection factors that were relevant to the outcomes of interest.

Covariates were slightly different than those used in the original paper in order to include additional potential confounders. [5]

### Statistical Analysis

Descriptive statistics were calculated to compare the covariate balance between customers at restaurants with and without labeling. Bivariate comparisons were made using chi-squared and t-tests. Multivariable linear regression models were used to examine mean differences in dietary outcomes between participants at labeled (treatment) and unlabeled (control) restaurants, controlling for the covariates listed above. Five separate models were used, one for each of the outcomes of interest. These results were compared to results from the propensity score methods described below.

#### Propensity scores

Propensity scores were calculated using a logistic regression model, with the outcome of patronizing a restaurant with labeling versus without. The covariates listed above were included as predictors. The resulting propensity score represented each participant’s predicted probability of patronizing a labeled restaurant based on the set of observed covariates. Matching was performed using the raw propensity scores, rather than using a logit transformation as has been used in some previous studies [18, 19], as this approach produced better covariate balance.

Propensity score analyses generally estimate either the average treatment effect (ATE) which represents the average effect of treatment on the whole study population; or the average treatment effect on the treated (ATT) which represents the average effect of treatment on those subjects who actually attended restaurants with menu labeling (the treated group). The estimand of interest in the present study was the ATT because in this study, as in many observational studies, the treated persons differed from untreated persons in significant ways. As such, estimating the ATT is more appropriate in this case.

#### Propensity Score Methods

After deriving each participant’s propensity score, the scores were used to match or weight treated and control individuals in preparation for the final outcome model. Four different methods were used. Matching was implemented using the MatchIt package in R (Methods 1 and 2) [20] and Matchit with the Optmatch package integrated (Method 3).[20, 21]

For Method 1 we selected the method most commonly used in studies of propensity scores,[16] greedy 1:1 nearest neighbor matching. This approach selects, for each individual in the treated group, the individual in the control group with the closest propensity score and matches one pair at a time[22]. “Greedy” matching indicates that when two treated individuals have the same control individual with the closest propensity score, the control will be matched to whichever treatment individual comes first in the matching order.[23] Once a match has been assigned, matches are not broken or revisited by the algorithm. Individuals in the control group who are not selected as a match for a treated individual are dropped from the matched dataset.

Method 2 augmented the 1:1 nearest neighbor matching with an additional exact match on race, a confounder that remained fairly imbalanced after matching, and a caliper of 0.2 standard deviations of the propensity score to ensure a closer match between treatment and control individuals. The caliper set a limit such that matched treated and control individuals’ propensity scores could not differ by greater than 0.2 standard deviations. A caliper of 0.2 has been previously described as an optimal width. [24] Due to the additional criteria placed on the match, this method would retain only about 70% of the study sample.

Method 3 was full matching,[18, 19, 23, 25] a lesser used approach that makes use of all observations in the data and has been found effective at reducing bias through producing very good covariate balance between treatment and comparison groups.[19] Full matching forms a series of matched subclasses where each set contains either at least one treated individual and multiple controls, or at least one control and multiple treated individuals. Observations are matched by minimizing a weighted average of the estimated distance measure between each treated subject and each control. In order to improve precision of the estimate and reduce dependence on any particular propensity score specification,[18, 26] we constrained the subclass ratios of treated to controls to be no less than 0.5 and no more than 2.0. This meant that within each matched subclass, the ratio of treated to controls was not fixed a priori but nevertheless would not vary widely. The range of 0.5 to 2.0 was chosen based on prior literature[18, 19] [the original sample ratio was approximately 1:1 (327 treated/321 control individuals)].

Finally, Method 4 was inverse probability weighting (IPW) by the propensity score.[16] IPW makes use of all observations in the dataset, is a widely used approach, and has been shown to provide good balance between groups.[27] In order to provide an appropriate comparison to the results obtained by matching methods, which inherently estimate the ATT rather than the ATE, we calculated each individual’s odds of treatment as weights in order to estimate the ATT.

Thus, individuals in the treatment group were assigned weights equal to 1, and individuals in the control group were assigned weights equal to [propensity score]/[1-propensity score].[28] This enables us to compare results from IPW to those obtained by matching methods, since by matching the control group to the treated group based on propensity scores, these methods inherently estimate the ATT rather than the ATE.

In order to evaluate the effectiveness of each method, balance was examined between the treated and untreated groups by calculating the standardized bias for each covariate in the propensity score model. The standardized bias is calculated by taking the difference in means for a given covariate between the treatment and control groups and dividing by the standard deviation in the treatment group. Standardized biases of less than 0.25 suggest good balance between the groups.[23] Boxplots were then constructed to display the absolute values of standardized biases before and after matching or weighting.[19]

After calculating the individual standardized biases for each covariate, the average standardized absolute mean distance (ASAM) was calculated for each method to provide a global comparison of the balance across methods. The ASAM is the absolute value of the standardized difference of means between the treatment and control groups (standardized by the standard deviation of each covariate in the treatment group), averaged across all of the covariates. A smaller ASAM indicates better balance between groups.

#### Determining the Effect of Menu Labeling on Nutritional Outcomes After Matching and Weighting

In order to determine whether measures of association between menu labeling and nutritional outcomes varied based on the propensity score method used, final outcome regression models were implemented using the matched or weighted data. For the nearest-neighbor methods (Methods 1 and 2), unweighted regression models were implemented using the reduced matched data set, adjusting for all covariates in the model in order to perform a doubly robust regression.[29] This is the same analysis as using the original sample but using the matched sample instead.

For the full matching analysis (Method 3), an ATT weighting approach was applied to the regression models in the full dataset. Within each matched set, each treated individual received a weight of 1 and controls received a weight proportional to the number of treated divided by the number of controls in that set.[23] As treated individuals receive a weight of 1, and control individuals are weighted to the treated individuals, this approach estimates the ATT. These weights were applied to linear regression outcome models that also controlled for the covariates of interest. For Method 4, the inverse probability weights (1 for the treated group, [propensity score]/[1-propensity score] for the control group) were applied to adjusted linear regression models.

All analyses were conducted using R version 3.0.3.[30] No identifiers were collected in the original study. The Drexel University Institutional Review Board approved the original study and determined that this retrospective secondary analysis of anonymous data did not qualify as human subjects research.

## Results

In total, 648 participants completed the survey (327 at restaurants with labeling, 321 at restaurants without labeling). Table 1 describes the balance of covariates between the two treatment groups. Customers at labeled restaurants were younger on average (mean 35 years old compared to 38 at unlabeled restaurants), a larger proportion was non-white, and a smaller proportion had income ≥$60,000. Customers at labeled sites were also less likely to dine out at least once per week, and to be enrolled on Sundays.

**Table 1:** Initial Covariate Balance between Treated (Labeled) and Control (Unlabeled) Restaurant Customers.

Variable	Labeled Restaurants N (%)	Unlabeled Restaurants N (%)	p-value^1^
N	327	321	
Age (mean, SD)	35.1 (13.0)	38.4 (14.0)	0.002
Sex			
Female	192 (59%)	199 (62%)	0.4
Male	135 (41%)	122 (38%)	
Race/Ethnicity			
White	100 (30%)	153 (48%)	<0.001
Black	182 (56%)	142 (44%)	
Hispanic and Other	45 (14%)	26 (8%)	
Income			
<$35,000	92 (28%)	65 (20%)	0.004
$35–60,000	101 (31%)	84 (26%)	
> = $60,000	134 (41%)	172 (54%)	
Education			
High School or Less	78 (24%)	79 (25%)	0.8
Technical or Associate’s Degree	51 (15%)	58 (18%)	
Bachelor’s Degree	133 (41%)	121 (38%)	
Graduate School	64 (20%)	63 (19%)	
Cautioned about Diet by Health Professional	70 (21%)	66 (21%)	0.8
Body Size			
Not severely overweight	291 (89%)	276 (86%)	0.3
Severely Overweight	36 (11%)	45 (14%)	
Frequency of Dining at Chain Restaurants			
> = Once per Week	123 (38%)	144 (45%)	0.06
<Once per Week	204 (62%)	177 (55%)	
Children in Party			
Yes	80 (24%)	75 (23%)	0.08
No	140 (43%)	184 (57%)	
Missing	107 (33%)	62 (20%)	
Customized Order	58 (18%)	64 (20%)	0.5
Day of the Week			
Sunday	16 (5%)	55 (17%)	<0.001
Tuesday	120 (37%)	74 (23%)	
Wednesday	92 (28%)	11 (35%)	
Thursday	99 (30%)	81 (25%)	

^1^P-values calculated by chi-squared tests

**Fig 1:**
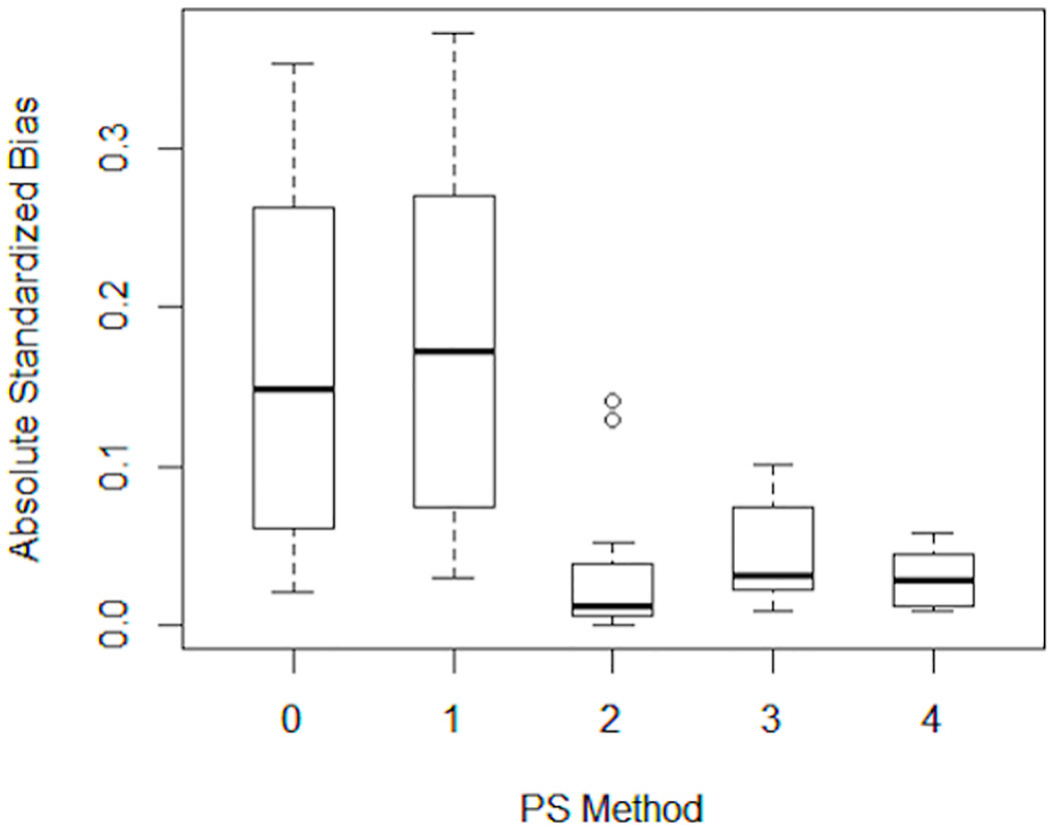
Boxplots of Absolute Standardized Biases for the Covariates in the Propensity Score Model. Methods. 0-original sample, no matching or weighting; 1- greedy 1:1 nearest neighbor matching, 2- greedy 1:1 nearest neighbor matching with exact match on race and caliper of 0.2, 3- full matching (constrained), 4- inverse probability weighting

Table 2 presents the covariate balance between participants at labeled and unlabeled restaurants before and after implementing the four propensity score methods. The standardized biases prior to matching reflect the imbalances shown in Table 1, with large standardized biases for age, race, income, day of the week, and number of children in the party. Method 1, greedy 1:1 nearest neighbor matching that was identical to the original sample except for 6 treated individuals who were not matched, resulted in worse overall balance than the original sample. Method 2, augmented 1:1 nearest neighbor matching, improved the balance between groups for most covariates, but resulted in the removal of 94 treated and 88 control individuals who did not have appropriately similar matches. Full matching (Method 3) and IPW (Method 4) resulted in reductions in the standardized biases for most covariates. Methods 2, 3 and 4 reduced the ASAM from the pre-matching level of 0.171, reflecting improved covariate balance. Fig 1 presents boxplots of the absolute values of the standardized biases for the covariates of interest, before and after implementing the four methods. Consistent with the results in Table 2, Methods 2, 3, and 4 resulted in absolute standardized biases that were closest to zero. Fig 1 and Table 2 together indicate that Method 4 (IPW) had the best bias reduction and uniformly good balance across all covariates.

**Table 2:** Comparison of Absolute Standardized Biases^a^ of Covariates between Treated (Labeled) and Control (Unlabeled) Subjects after Propensity Score Matching and Weighting^b^.

Variable	Unmatched Sample	Method 1. Greedy 1:1 Nearest Neighbor Matching	Method 2. Greedy Nearest Neighbor + Exact Match + Caliper of 0.2 SD	Method 3. Full Matching, Constrained	Method 4. Inverse Probability Weighting^d^
Age	0.253*	0.266*	0.012	0.072	0.010
Sex	0.067	0.076	0.009	0.022	0.011
Race	0.353*	0.372*	0.000	0.076	0.029
Income	0.249	0.269*	0.026	0.074	0.042
Education	0.040	0.050	0.004	0.013	0.014
Body Size	0.096	0.119	0.027	0.083	0.059
Cautioned about Diet	0.021	0.030	0.052	0.030	0.047
Frequency of dining out	0.149	0.173	0.142	0.032	0.011
Day of the week	0.272*	0.271*	0.004	0.010	0.053
Customizations	0.056	0.073	0.011	0.024.	0.034
Children in Party	0.323*	0.335*	0.129	0.101	0.026
Average Standardized Absolute Mean Difference (ASAM)^c^	0.171	0.185	0.038	0.049	0.031
N Treated/N Discarded	327	321/6	233/94	327/0	327/0
N Control/N Discarded	321	321/0	233/88	321/0	321/0

^a^Absolute standardized bias is the absolute value of the weighted difference in means between the treatment and control group divided by the standard deviation in the treatment (labeled) group.

^b^Propensity scores were calculated using logistic regression.

^c^The ASAM is calculated by taking the average of the absolute values of the standardized biases for all covariates used to calculate the propensity score (not including the propensity score itself).

^d^Inverse probability weights indicate odds of being in the treatment (labeled) group and were calculated as follows: 1 for treated, [propensity score]/[1-propensity score] for control

*indicates standardized bias >0.25

The results of the linear regression models assessing the association of menu labeling with calories, saturated fat, carbohydrates, and sodium purchased, before and after matching, are presented in Table 3. In the pre-match, unweighted models, menu labeling was associated with a statistically significant reduction in total calories purchased (-179.6), food calories (-178.2), saturated fat (-46), carbohydrates (-16.1), and sodium (-279.4). In models accounting for each of the four propensity score methods, results were substantively similar but slightly attenuated for most outcomes. Specifically, menu labeling was associated with a reduction in total calories of -149.0 and -135.3 for constrained full matching and IPW, respectively, although these reductions remained statistically significant. The same pattern was observed for food calories (-149.7 and -139.1 for full matching and IPW). The only outcome that was no longer significantly associated with menu labeling after using a propensity score-based method was saturated fat using Method 2 (nearest neighbor matching with a caliper and exact match on race), which was -3.3 (-7.6, 0.9). All associations were in the same direction as in the initial models.

**Table 3:** Effect of Menu Labeling on Nutritional Outcomes^a^ Before and After Propensity Score Matching/Weighting^b^.

	Original Adjusted Regression Models	Method 1. Greedy 1:1 Nearest Neighbor Matching	Method 2. Greedy Nearest Neighbor + Exact Match on Race + Caliper of 0.2 SD	Method 3. Full Matching, Constrained	Method 4. Inverse Probability Weighting^c^
Total Calories (kcal)	-179.6 (-310.0, -49.2)*	-165.3 (-297.8, -32.8)*	-167.7 (-314.3, -21.2)*	-149.0 (-273.4, -24.6)*	-135.3 (-260.0, -10.6)*
Food Calories (kcal)	-178.2 (-299.3, -57.1)*	-161.4 (-284.3, -38.4)*	-167.9 (-305.8, -30.1)*	-149.7 (-264.4, -34.9)*	-139.1 (-255.6, -22.5)*
Food Saturated Fat (g)	-4.6 (-8.4, -0.9)*	-4.3 (-8.0, -0.5)*	-3.3 (-7.6, 0.9)	-3.6 (-7.1, 0.0)*	-4.5 (-8.2, -0.7)*
Food Carbohydrates (g)	-16.1 (-27.5, -4.7)*	-14.8 (-26.4, -3.3)*	-15.8 (-29.0, -2.6)*	-16.6 (-27.5, -5.6)*	-14.7 (-25.7, -3.8)*
Food Sodium (mg)	-279.4 (-515.5, -43.4)*	-252.5 (-492.0, -12.9)*	-338.2 (-608.8, -67.5)*	-282.5 (-512.9, -52.1)	-212.4 (-440.4, 15.7)

^a^Each model presents the mean difference in the nutrition outcome in question between customers at labeled restaurants and customers at unlabeled restaurants, adjusted for the following covariates: age, sex, race/ethnicity, income, education, body size, whether they were cautioned about their diet, frequency of dining out, day of the week, whether there were children in the party, and whether the order was customized.

^b^Results reported here differ from regression models reported in Auchincloss et al (2013) due to (a) adding three additional covariates (whether a health professional cautioned the participant about his/her diet, whether there were children in the party, and whether the customer made any substitutions or customizations to their order) as covariates to the unweighted model and (b) estimating the ATT in the Inverse Probability weighted regression models rather than the ATE. The original paper reported that the treatment difference in overall calories was -155.0 (-284.0, -27.0) in the unweighted model and -166.6 (-286.7, -46.5) in the weighted model.

*indicates p<0.05

^c^Inverse probability weights indicate odds of being in the treatment (labeled) group and were calculated as follows: 1 for treated, [propensity score]/[1-propensity score] for control

## Discussion

In this study of a menu labeling natural experiment, the initial imbalance in characteristics between customers at labeled and unlabeled restaurants was improved by three out of four propensity score matching methods. Of these methods, constrained full matching and IPW resulted in a substantial reduction in standardized bias without a reduction in sample size. The association between menu labeling and nutritional outcomes was fairly stable across methods and was slightly attenuated, though substantively similar, compared to the initial unmatched/unweighted regression models. These findings indicate that good balance can be achieved using several methods. In addition, our findings strengthen inferences about the association between menu labeling at sit-down restaurants and reduction in calories, saturated fat, carbohydrates, and sodium purchased, as the results obtained after making the treatment and control group more comparable were substantively similar to those observed in the original analysis published from this study.[5]

Researchers are increasingly turning to propensity scores as a tool to improve causal inference. However, many methods are available and choosing the right method can be challenging. 1:1 nearest neighbor matching on the propensity score is one of the most common methods;[26] however, we found that this form of matching did not improve the standardized bias in this study, as the matched sample was very similar to the original sample and no weights were applied. Augmenting the match with a caliper, a method that has been suggested previously to improve the quality of the matching process,[16] and an exact match on an unbalanced covariate resulted in a very close match but substantially reduced the sample size, an important consideration to keep in mind when implementing these methods. Loss of power is a possible limitation of imposing such strict criteria on the matching, as only ~70% of the study population was retained. In addition, 94 treated individuals were discarded. Loss of treated individuals may shift the estimation target as the treatment effect may no longer represent the effect for all participants in the treatment group.[31]

In consideration of these limitations, our findings suggest that researchers may benefit from several lesser-used but easy–to-implement methods, namely full matching and IPW. Full matching, which has been described as a compromise between k:1 matching and subclassification,[19] is more flexible than nearest neighbor matching because the number of comparison individuals matched to each treated individual can vary depending on how many good matches are actually available. Full matching also makes use of all available data (does not drop any individuals from the analysis), and has been found to perform better than k:1 matching in simulated and real data.[19, 32] Our finding that constrained full matching resulted in an overall reduction in standardized bias is consistent with a prior study comparing various methods of matching in a cross-sectional study of the relationship between adolescent marijuana use and adult outcomes.[19] Finally, IPW performed quite well in terms of improving the balance in this study; this finding is consistent with prior research.[27] These results highlight the usefulness of implementing several different methods and comparing to ensure results are consistent, as we found in this comparative analysis.

Propensity score methods have been used in previous natural experiment studies of several built environment or policy-based interventions, including park renovations,[15] public transit infrastructure,[13] and a pay-for-performance program for diabetes care.[14] Methods have included weighting[15, 33, 34] and matching.[13, 14] However, these approaches are underutilized. Many previous evaluations of menu labeling policies have used multivariable linear regression models to control for differences between treated and control groups;[6–9, 35] however, regression relies heavily on modeling assumptions regarding the functional form of the relationship between the covariates and the outcome and may be problematic if there are large differences between the two groups.[36] Using IPW, full matching, or other propensity score-based approaches in tandem with multivariable regression may be beneficial in such studies for ensuring that the treatment and control groups are as similar as possible in order to strengthen causal claims.

This study had several limitations. First, the study included a convenience sample of treatment and control restaurant outlets in both urban and suburban areas; however, all data were collected from the same restaurant chain in one part of the U.S. during a single month, which limited variation in potential restaurant-level confounders such as price, menu offerings, and restaurant marketing and promotions. Additionally, the propensity score-based analyses relied on the assumption that there were no unobserved confounding variables, which could be more fully evaluated using a sensitivity analysis. However, we included a comprehensive list of potential confounders in the analyses. Propensity score matching on observed covariates also matches on unobserved covariates that are correlated with observed covariates, but would not account for imbalance in other unobserved covariates.

## Conclusion

In this menu labeling natural experiment, full matching on propensity scores and inverse probability weighting were useful methods for improving balance between study groups without a reduction in sample size. The association between menu labeling and purchasing restaurant items lower in calories, fat, carbohydrates, and sodium was slightly attenuated when compared to results that did not use propensity score methods, but results were substantively similar.
